# Corticosterone enhances the potency of ethanol against hippocampal long-term potentiation via local neurosteroid synthesis

**DOI:** 10.3389/fncel.2015.00254

**Published:** 2015-07-03

**Authors:** Yukitoshi Izumi, Kazuko A. O’Dell, Charles F. Zorumski

**Affiliations:** Department of Psychiatry, Taylor Family Institute for Innovative Psychiatric Research, Washington University School of MedicineSt. Louis, MO, USA

**Keywords:** stress, corticosterone, binge drinking, blackout, LTP, allopregnanolone

## Abstract

Corticosterone is known to accumulate in brain after various stressors including alcohol intoxication. Just as severe alcohol intoxication is typically required to impair memory formation only high concentrations of ethanol (60 mM) acutely inhibit long-term potentiation (LTP), a cellular memory mechanism, in naïve hippocampal slices. This LTP inhibition involves synthesis of neurosteroids, including allopregnanolone, and appears to involve a form of cellular stress. In the CA1 region of rat hippocampal slices, we examined whether a lower concentration of ethanol (20 mM) inhibits LTP in the presence of corticosterone, a stress-related modulator, and whether corticosterone stimulates local neurosteroid synthesis. Although low micromolar corticosterone alone did not inhibit LTP induction, we found that 20 mM ethanol inhibited LTP in the presence of corticosterone. At 20 mM, ethanol alone did not stimulate neurosteroid synthesis or inhibit LTP. LTP inhibition by corticosterone plus ethanol was blocked by finasteride, an inhibitor of 5α-reductase, suggesting a role for neurosteroid synthesis. We also found that corticosterone alone enhanced neurosteroid immunostaining in CA1 pyramidal neurons and that this immunostaining was further augmented by 20 mM ethanol. The enhanced neurosteroid staining was blocked by finasteride and the N-methyl-D-aspartate antagonist, 2-amino-5-phosphonovalerate (APV). These results indicate that corticosterone promotes neurosteroid synthesis in hippocampal pyramidal neurons and can participate in ethanol-mediated synaptic dysfunction even at moderate ethanol levels. These effects may contribute to the influence of stress on alcohol-induced cognitive impairment.

## Introduction

Plasma levels of corticosterone, the major glucocorticoid in mice and rats, are increased by various types of stress, including alcohol intoxication. The rise in plasma corticosterone can result in increased corticosterone levels in brain (Chauveau et al., 2010), where corticosterone activates glucocorticoid receptors (GRs) and mineralocorticoid receptors (MRs). GRs are densely expressed in the hippocampus and MRs are found in the hippocampus and septum (Reul and de Kloet, 1985; Herman et al., 1989; Herman and Spencer, 1998).

Long-term potentiation (LTP) in the hippocampus is a cellular mechanism thought to underlie memory formation, and numerous studies have shown that stress can acutely and chronically impair both memory acquisition and LTP induction (Foy et al., 1987; Shors et al., 1989; Tabassum and Frey, 2013). For these reasons, it has been thought that increases in corticosterone levels in the hippocampus mediate LTP inhibition, and thus impair memory processing. Indeed, prolonged corticosterone administration disturbs memory acquisition (Bodnoff et al., 1995), and 2 or 3 h administration of 1 μM corticosterone inhibits LTP induction in rats (Zhou et al., 2000; Park et al., 2015). However, LTP inhibition by corticosterone is conditional and 1 h administration of 1 μM corticosterone does not inhibit LTP induction, although it may alter the magnitude of synaptic enhancement (Maggio and Segal, 2007). The effects of corticosterone are also dose dependent (Diamond et al., 1992). In acute murine hippocampal slices, 5 μM corticosterone reduces while 0.5 nM corticosterone facilitates LTP (Rey et al., 1994). In the CA1-subiculum region, corticosterone alone does not mimic the effects of acute stress in inhibiting LTP induction (MacDougall and Howland, 2013). Adding to the complexity (Joëls, 2006), corticosterone can promote activation of NMDA-type glutamate receptors (Joëls and Krugers, 2007) and alter the trafficking of AMPA-type glutamate receptors (Martin et al., 2009). Corticosterone can also foster glutamate accumulation by effects on glutamate uptake (Sandi, 2011). Furthermore, repeated pulses of corticosterone, mimicking daily patterns of release, can acutely enhance glutamate transmission, but subsequently renormalize plasticity (Sarabdjitsingh et al., 2014) These results suggest that corticosterone has complex effects on hippocampal function and may play a permissive, but as yet incompletely defined role in modulating LTP and memory during acute stress.

Neurosteroids such as allopregnanolone (AlloP) that enhance the function of γ-aminobutyric acid-A receptors (GABA_A_Rs) modulate LTP and learning after various stressors (Zorumski and Izumi, 2012; Zorumski et al., 2014). In particular, ethanol administration impairs memory acquisition through AlloP production (Barbaccia et al., 1999; Morrow et al., 1999, 2006; VanDoren et al., 2000; Matthews et al., 2002). In rat hippocampal slices high concentrations of ethanol (50–60 mM) facilitate local AlloP synthesis (Follesa et al., 2006; Tokuda et al., 2011) and inhibit LTP induction (Izumi et al., 2005, 2007). In contrast to the effects of 60 mM ethanol, a lower concentration of ethanol (20 mM) does not facilitate AlloP production (Tokuda et al., 2011) and does not block LTP, although LTP is inhibited when 20 mM ethanol is combined with exogenously administered AlloP (Izumi et al., 2007). Furthermore, 20 mM ethanol inhibits LTP induction when combined with acetaldehyde, an agent that facilitates AlloP production in pyramidal neurons (Tokuda et al., 2013). AlloP synthesis is enhanced by various stressors and drugs, and contributes to LTP inhibition (Tokuda et al., 2010; Izumi et al., 2013). Whether corticosterone plays a role in mediating the effects of stress on neurosteroid production remains unknown. In the present study, we examined whether exogenous corticosterone acutely alters LTP and neurosteroid production alone and in the presence of ethanol in rat hippocampal slices.

## Materials and Methods

### Hippocampal Slice Preparation

All methods in this paper have been described previously in our publications and are repeated briefly here (Tokuda et al., 2010, 2011). Animal use followed NIH guidelines and was approved by the Washington University Animal Studies Committee. We prepared hippocampal slices from postnatal day (P) 30–32 albino rats (Tokuda et al., 2010). To avoid problems with circadian rhythms and cortisol levels, slices were prepared at about 11 AM on the day of an experiment. Under isoflurane anesthesia, rats were decapitated. Dissected hippocampi were pinned at their ventral pole on a 3.3% agar base in ice-cold artificial cerebrospinal fluid (ACSF) containing (in mM): 124 NaCl, 5 KCl, 2 MgSO_4_, 2 CaCl_2_, 1.25 NaH_2_PO_4_, 22 NaHCO_3_, 10 glucose, bubbled with 95% O_2–_5% CO_2_ at 4–6°C. The dorsal two-third section of the hippocampus was cut into 500 μm slices using a rotary slicer. Acutely prepared slices were placed in an incubation chamber containing gassed ACSF for at least 1 h at 30°C before further study.

### Hippocampal Slice Physiology

For physiology studies, slices were moved to a submersion-recording chamber at 30°C with ACSF perfused at 2 ml/min. Extracellular recordings were obtained from the apical dendritic layer (*stratum radiatum*) of the CA1 region for recording excitatory postsynaptic potentials (EPSPs) with electrodes filled with 2 M NaCl (5–10 MΩ resistance).

**Figure 1:**
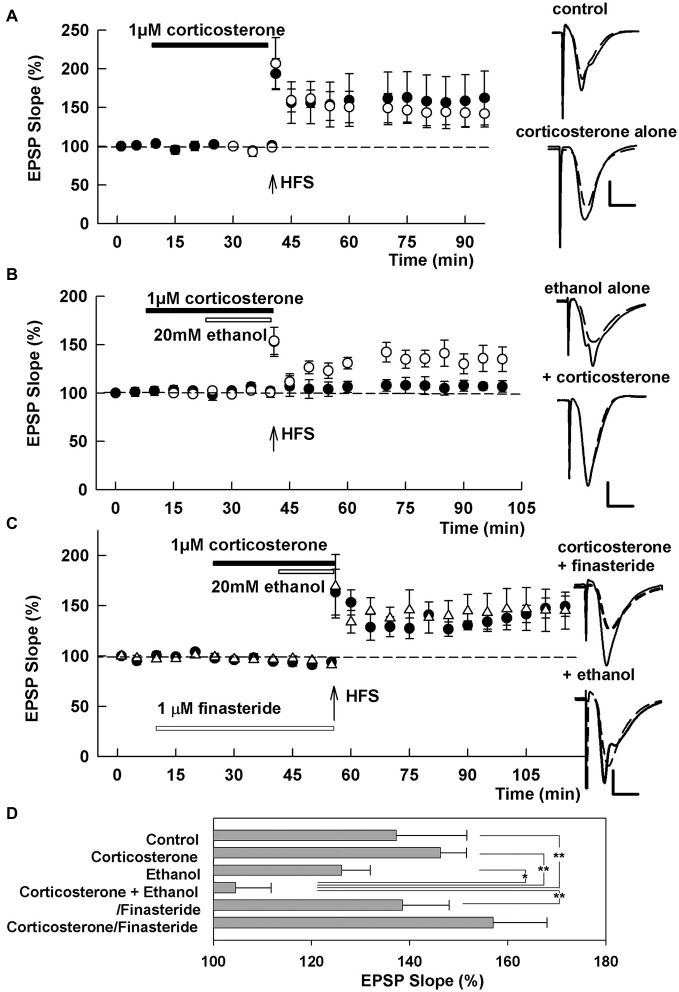
**Effects of corticosterone and ethanol on long-term potentiation (LTP). (A)** LTP is readily induced by a single 100 Hz × 1 s high frequency stimulation (HFS; arrow) in control slices (open circles) and even in the presence of 1 μM corticosterone (closed bar, closed circles). **(B)** LTP is not blocked by 20 mM ethanol (open bar, open circles), but is blocked by a combination of 20 mM ethanol and 1 μM corticosterone with corticosterone applied for 15 min before and during ethanol administration (closed circles). **(C)** The inhibition of LTP by 20 mM ethanol and 1 μM corticosterone is overcome by pretreatment of slices with 1 μM finasteride. Traces depict excitatory postsynaptic potentials (EPSPs) before (dash lines) and 60 min after HFS. Scale: 1 mV, 5 ms. **(D)** The graph shows a summary of results under the various conditions 60 min following HFS. P-values are calculated with Student *t*-test (*P < 0.05, **P < 0.01).

EPSPs were evoked with 0.1 ms constant current pulses through a bipolar stimulating electrode in the Schaffer collateral (SC) pathway. Responses were monitored by applying single stimuli to the SC pathway every 60 s at half maximal intensity. After obtaining a control input-output curve and stable baseline recordings for 10 min, LTP was induced by a single 100 Hz × 1 s high frequency stimulation (HFS) at the same intensity stimulus. Input-output curves were repeated 60 min following HFS. In some experiments an IO curve was also repeated 20 min after HFS and appears as a gap in the graphs in Figures 1A,B.

### Immunohistochemistry

Slices for immunohistochemistry were screened for electrophysiological responses and were incubated with various reagents in separate 10 ml beakers as previously described (Tokuda et al., 2010). Slices selected for immunochemistry displayed paired-pulse enhancement of EPSPs, a sign of robust slice health under the recording conditions used (Tokuda et al., 2010). After drug treatment, slices were fixed in phosphate buffered saline (PBS) with four percent paraformaldehyde (PFA) n for 30 min, washed with PBS and incubated in blocking solution (one percent donkey serum/PBS) for 2 h at 25°C. Slices were then incubated without permeabilization in an antibody raised in sheep against 5α-reduced neurosteroids diluted 1:2500 in 1% donkey serum/PBS for 48 h at 4°C then rinsed with PBS and incubated with secondary antibody for 2 h at 25°C. Alexa Fluor 488 donkey anti-sheep IgG (diluted 1:500) was used for neurosteroid visualization. In some experiments nuclei were stained with 4, 6-diamidino-2-phenylindole (Dapi) for 15 min at room temperature. High magnification microscopic imaging was performed using a C1 laser scanning confocal microscope with 40× objective (1.4 N.A.), and digitized with Z-C1 software (Nikon Instruments, Melville, NY, USA), or with a Zeiss Axoimage Z1with 20× objective (0.8 DIC II) and digitized using ImageJ. Images were taken in z-stacks of 0.4 μM steps. All acquisition parameters were kept constant within an experiment. Digital images were analyzed and the average intensity of the tissue was measured using MetaMorph software (Universal Imaging Corporation, Downingtown, PA, USA).

### Chemicals

Anti-AlloP antiserum was purchased from the late Robert Purdy, University of California-San Diego. Alexa Fluor 488 was purchased from Invitrogen (Carlsbad, CA, USA). Finasteride was purchased from Steraloids (Newport, RI, USA). All other chemicals were purchased from Sigma Chemical Company (St. Louis, MO, USA). Finasteride and corticosterone were prepared as stock solutions in ethanol. Drugs were dissolved in ACSF at the time of experiment and administered by bath perfusion at the concentrations noted in the text. The concentrations selected for study and the durations of drug administration were based on prior studies examining their effects on synaptic transmission and synaptic plasticity in the absence of effects of baseline transmission in naïve slices.

### Statistical Analysis

Data were collected and analyzed using PClamp software (Axon Instruments, Union City, CA, USA). Data are expressed as mean ± SEM 60 min following HFS, and are normalized with respect to initial baseline recordings (taken as 100%). A two-tailed Student’s *t*-test was used for comparisons between groups. In cases of non-normally distributed data, the non-parametric Wilcoxon Rank Sum Test was used. Additionally, analysis of variance (ANOVA) was performed on between group means. Statistical comparisons were based on input-output curves at baseline and 60 min following HFS to determine the degree of change in EPSP slope at the 50% maximal point with *p* < 0.05 considered significant. Statistics were performed using commercial software (SigmaStat, Systat Software, Inc., Richmond City, CA, USA).

## Results

### LTP Inhibition by 20 mM Ethanol Plus Corticosterone

We initially examined the effects of acute corticosterone alone on LTP induction. Thirty minute administration of 1 μM corticosterone did not inhibit LTP induction produced by a single 100 Hz × 1 s HFS when perfused prior to and during HFS (EPSP slopes 60 min after HFS: 146.3 ± 14.4%, *N* = 5, closed circles in Figure 1A), although there was some slice-to-slice variability in the overall magnitude of LTP. Similarly, 10 μM corticosterone alone did not inhibit LTP induction (128.9 ± 9.4%, *N* = 3, data not shown). The degree of LTP in the presence of corticosterone did not differ significantly from control LTP in naïve slices (137.3 ± 5.3%, *N* = 5, open circles in Figure 1A). In prior studies, we found that 20 mM ethanol alone does not acutely inhibit LTP induction (Izumi et al., 2007; Tokuda et al., 2011). Consistent with this, 20 mM ethanol did not inhibit LTP in the present study (126.2 ± 5.8%, *N* = 5, open circles in Figure 1B). However, a combination of 20 mM ethanol and 1 μM corticosterone blocked LTP induction completely (104.6 ± 14.4%, *N* = 6, closed circles in Figure 1B). For these studies, corticosterone was administered for 15 min prior to and during a 15 min ethanol perfusion.

Because LTP inhibition by 60 mM ethanol involves local neurosteroid synthesis (Izumi et al., 2007; Tokuda et al., 2011), we also examined whether the effects of corticosterone plus 20 mM ethanol were altered by finasteride, a specific inhibitor of 5α reductase, a key enzyme in neurosteroid synthesis. When slices were pretreated with 1 μM finasteride for 15 min prior to other drugs, the combination of corticosterone plus ethanol failed to inhibit LTP induction (138.6 ± 9.5%, *N* = 5, Figure 1C), suggesting that corticosterone may promote LTP inhibition via de novo neurosteroid synthesis in the hippocampus. Finasteride pretreatment also did not alter LTP in the presence of corticosterone alone (157.1 ± 10.9%, *N* = 5, Figure 1C). A summary of results from the LTP experiments based on analysis of IO curves 60 min following HFS is shown in Figure 1D (*p* = 0.006 by one-way ANOVA).

### Neurosteroid Synthesis by 20 mM Ethanol Plus Corticosterone

**Figure 2:**
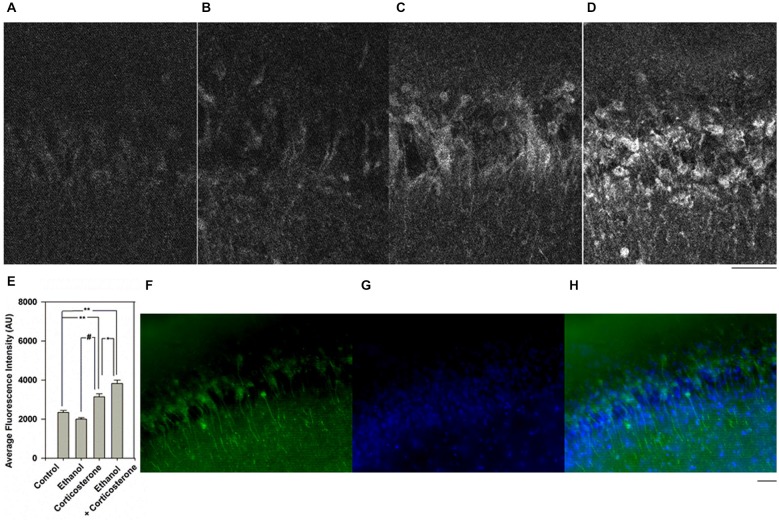
**Effects of corticosterone and ethanol on neurosteroidogenesis. (A)** Immunostaining against 5-alpha-reduced neurosteroids in the CA1 area in a naïve control hippocampal slice. **(B)** Neurosteroid immunostaining is not enhanced by 30 min administration of 20 mM ethanol. **(C)** Immunostaining is enhanced when slices are incubated with 1 μM corticosterone for 30 min. **(D)** Immunostaining is further enhanced when slices are incubated with 20 mM ethanol plus 1 μM corticosterone for 30 min. Note that in these experiments, corticosterone and ethanol were co-administered for 30 min. **(E)** Summary graph showing staining intensity in arbitrary units. P-values are calculated with Student *t*-test (*P < 0.05, **P < 0.01) or by Mann-Whitney U-test (^#^*P* < 0.05, *n* = 4). **(F–H)** Dapi staining (blue) and immunostaining (green) against 5-alpha-reduced neurosteroids in the CA1 area in a slice treated with 1 μM corticosterone and 20 mM ethanol. Scale: 25 μm.

We subsequently determined whether corticosterone promotes local neurosteroid synthesis by immunostaining in the CA1 region. For these studies we used an antibody against 5α-reduced neurosteroids which we have previously characterized to detect changes in local neurosteroid production following ethanol, benzodiazepines and stressors (Tokuda et al., 2010, 2011). Consistent with our prior results (Tokuda et al., 2011), 20 mM ethanol alone did not promote neurosteroid synthesis (Figures 2A,B). In contrast, administration of 1 μM corticosterone increased neurosteroid staining compared to control (Figure 2C, N = 4 for each condition; *P* < 0.01 vs. control by Student’s *t*-test). A combination of 20 mM ethanol with corticosterone robustly enhanced neurosteroid immunostaining (Figure 2D), with the degree of enhancement with the combination of corticosterone and ethanol being significantly greater than corticosterone alone (Figure 2E; *P* < 0.05, *N* = 4 by Student’s *t*-test). A summary statistical analysis is shown in Figure 2E (*p* < 0.001 by one-way ANOVA). Cells in the pyramidal cell layer were positive with Dapi staining (Figure 2F) and showed partial overlap with neurons co-immunostained using the antibody against 5α-reduced neurosteroids (Figures 2G,H).

**Figure 3:**
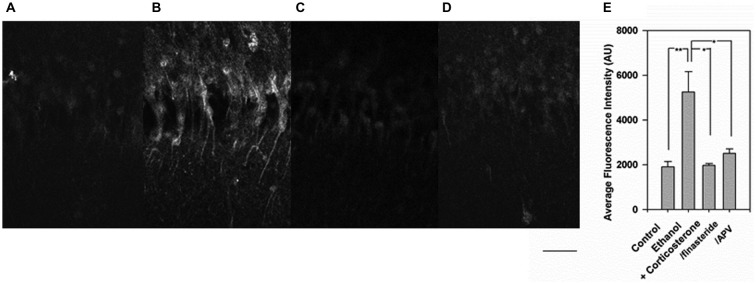
**Inhibition of the enhanced neurosteroidogenesis with corticosterone and ethanol by finasteride and 2-amino-5-phosphonovalerate (APV). (A)** Immunostaining against 5-alpha-reduced neurosteroids in the CA1 area in a naïve control hippocampal slice. **(B)** Immunostaining is enhanced when slices are incubated with 1 μM corticosterone for 30 min. **(C)** The enhancement of neurosteroid immunostaining is overcome by pretreatment of slices with 1 μM finasteride. **(D)** Similarly, the enhancement of neurosteroid immunostaining is overcome by pretreatment of slices with 100 μM APV. **(E)** Summary graph showing staining intensity in arbitrary units. P-values are calculated with Student *t*-test (*P < 0.05, **P < 0.01, *n* = 4). Scale: 25 μm.

To address possible mechanisms contributing to the effects of corticosterone and ethanol on neurosteroid synthesis, slices were pretreated with 1 μM finasteride or 100 μM D, L-amino-5-phosphonovalerate (APV) before administration of 1 μM corticosterone and 20 mM ethanol in a separate set of experiments. Again, the combination of corticosteroid and 20 mM ethanol enhanced immunostaining against 5α-reduced neurosteroids (Figure 3, *N* = 4 for each condition). The enhancement was clearly suppressed by both finasteride (Figure 3C) and APV (Figure 3D). A summary of results with statistics is shown in Figure 3D (*p* = 0.003 by one-way ANOVA).

## Discussion

Various chemicals and drugs including ammonia (Izumi et al., 2013), acetaldehyde (Tokuda et al., 2013) and midazolam (Tokuda et al., 2010) promote local neurosteroid synthesis in the hippocampus. Ethanol also enhances neurosteroid local synthesis at high concentrations (Sanna et al., 2004; Follesa et al., 2006). In hippocampal slices, 60 mM ethanol acutely promotes neurosteroid synthesis (Tokuda et al., 2011) and impairs LTP induction (Izumi et al., 2007). Ethanol does not inhibit LTP at lower concentrations that do not promote neurosteroid synthesis (Tokuda et al., 2011). Although 20 mM ethanol alone does not inhibit LTP induction, it markedly impairs LTP if combined with exogenous AlloP. This finding suggests that even moderate alcohol consumption may adversely affect cognitive function under conditions in which neurosteroid synthesis is enhanced. Because it is known that corticosterone is released during and after stressful events, and because corticosterone is thought to be synthesized exclusively in the adrenal cortex, we hypothesized that corticosterone may enter the brain and trigger neurosteroid synthesis to alter memory processing. If this is the case, brain neurosteroid synthesis will be enhanced under conditions when stressful events trigger sufficient peripheral corticosterone production.

Numerous studies have shown that acute and chronic stressors can suppress LTP (Foy et al., 1987; Shors et al., 1989; Diamond et al., 1992), and that stressful events increase corticosterone levels in the brain. For example, training in the water maze increases plasma corticosterone levels and dampens LTP (Tabassum and Frey, 2013), and direct application of corticosterone can inhibit LTP induction (Zhou et al., 2000). In our study, however, even a high (1–10 μM) concentration of corticosterone alone failed to inhibit LTP induction acutely in hippocampal slices during a 30 min administration. This result suggests that other factors, in addition to exogenously administered corticosterone, are critical for LTP inhibition. Consistent with this, LTP was inhibited after acute stress in the subiculum of anesthetized rats, but this effect was not mimicked by corticosterone injection (MacDougall and Howland, 2013). Of note, low micromolar corticosterone has been found to dampen LTP when administered for two or more hours (Park et al., 2015), as might occur under conditions of extreme stress.

In our study, we found that exogenous corticosterone promotes LTP inhibition in the presence of 20 mM ethanol, but that neither agent was effective against LTP at the concentrations studied. This finding suggests that even more moderate alcohol consumption, in the absence of profound intoxication, may alter memory acquisition when corticosterone synthesis is increased by other stressors. Because the effects on LTP are blocked by finasteride, this LTP inhibition involves the synthesis of 5α-reduced neurosteroids including AlloP. Consistent with this, we found that corticosterone enhanced local neurosteroid immunostaining in the CA1 region. However, as we have shown previously, increases in neurosteroid levels alone are not sufficient to block LTP induction (Tokuda et al., 2010) This observation has led us to speculate that LTP inhibition in the presence of elevated neurosteroids requires a second and more variable factor (Zorumski and Izumi, 2012). In the case of amnesic benzodiazepines, the second factor is active at the benzodiazepine site on GABA-A receptors leading to an increase in proximal inhibition in the CA1 region (Tokuda et al., 2010). Neither effect alone, benzodiazepine site activity or neurosteroid production, is sufficient for benzodiazepines to block LTP induction. In the case of ethanol, partial NMDA receptor antagonism in the face of ongoing glutamate release and/or accumulation is likely to be critical, leading to activation of unblocked NMDA receptors and activation of intracellular signaling that includes serine phosphatases, nitric oxide synthase and p38 mitogen activated protein kinase (Izumi et al., 2008). Interestingly, the combination of corticosterone and 20 mM ethanol exacerbated neurosteroid immunostaining even though 20 mM ethanol alone did not enhance steroid staining (Tokuda et al., 2011). The enhancement was observed in the soma and major dendrites of pyramidal cells, but we note that all cells in the *stratum pyramidale* were not positive for 5 α-reduced neurosteroids. We have previously observed that only some neurons in the pyramidal cell layer are immunopositive for neurosteroids when exposed to high ethanol or low micromolar NMDA (Tokuda et al., 2010, 2011).

The present study begins to provide information about mechanisms contributing to neurosteroid production in the presence of corticosterone and low ethanol. In prior work, we found that the ability of 60 mM ethanol to enhance neurosteroid production and to inhibit LTP was overcome by an NMDA receptor antagonist administered during the period of ethanol exposure (Tokuda et al., 2011). Because other studies indicate that corticosterone can promote glutamate accumulation, glutamate transmission (Joëls, 2006; Joëls and Krugers, 2007) and NMDA receptor activation, perhaps through effects on glutamate transport (Sandi, 2011), we examined a role for NMDA receptors in the effects of corticosterone plus ethanol. Just as we observed with 60 mM ethanol, we found that enhanced neurosteroid staining in the CA1 region was blocked by co-administration of the broad spectrum NMDA receptor antagonist, APV. This strongly suggests that accumulation of glutamate or another agonist activates NMDA receptors that are left unblocked by ethanol to drive neurosteroid production (Zorumski and Izumi, 2012). How NMDA receptors produce their effects on neurosteroids is uncertain, but other work indicates that calcium influx (Capponi et al., 1988; Rossier, 2006), including influx via NMDA receptors (Kimoto et al., 2001), can stimulate neurosteroid synthesis. Thus calcium influx via NMDA receptors can drive both local steroid synthesis and metaplastic LTP inhibition in the hippocampus (Izumi et al., 1992).

While we do not understand how ethanol and corticosterone interact in the hippocampus, the enhanced response in the presence of both agents could involve local production of corticosteroid or another messenger. Although it is unclear whether the brain can synthesize corticosterone locally, and prior work using ELISA measurements report the loss of corticosterone in rat brains after adrenalectomy (Gomez-Sanchez et al., 2005), a recent study using higher resolution LC-MS/MS methods detected corticosterone in rat hippocampus after adrenalectomy (Higo et al., 2011). This latter study also demonstrated the expression of p450c21, a critical enzyme for corticosterone synthesis, in the hippocampus. If corticosterone plus ethanol triggers de novo synthesis of endogenous corticosterone in the hippocampus, this might result in enhanced stimulation of neurosteroid synthesis. It is also possible that ethanol alone can stimulate endogenous corticosterone synthesis in the hippocampus, and corticosterone levels have been found to be very high in the hippocampus after ethanol injection (Croft et al., 2008). Again, our prior studies indicated that LTP inhibition and neurosteroid production by high concentrations of ethanol involve a form of NMDA receptor-mediated metaplasticity (Zorumski and Izumi, 2012), and this is consistent with our finding that enhanced neurosteroid synthesis by corticosterone and low ethanol is blocked by an NMDA receptor antagonist. Thus, interactions among corticosterone, ethanol and glutamate may be critical for the effects observed in the present studies. Future studies are needed to address these interactions and whether corticosterone is intrinsically synthesized in the hippocampus.

## Author Contributions

YI and CZ designed the studies. YI and KO performed the experiments. YI and CZ analyzed data and wrote the manuscript. All authors approved the final manuscript.

## Conflict of Interest Statement

CZ serves on the Scientific Advisory Board of Sage Therapeutics. The authors declare that the research was conducted in the absence of any commercial or financial relationships that could be construed as a potential conflict of interest.
